# Psychometric Properties of the Dutch Surrender to God Scale: Relationships with Religious Behavior, God Representation, Well-being, and Health

**DOI:** 10.1007/s10943-024-02144-y

**Published:** 2024-10-04

**Authors:** Henk-Jan Seesink, Michelle van Dusseldorp, Brian D. Ostafin, Hanneke Schaap-Jonker, Reinout W. Wiers

**Affiliations:** 1 Research Department, De Hoop GGZ, Provincialeweg 70, 3329 KP Dordrecht, The Netherlands; 2 Addiction Development and Psychopathology (ADAPT)-Lab, Department of Psychology, University of Amsterdam, Amsterdam, The Netherlands; 3 Centre for Research and Innovation in Christian Mental Health Care, Eleos/De Hoop, Hoevelaken, The Netherlands; 4 Experimental and Clinical Psychology, Department of Psychology, University of Groningen, Groningen, The Netherlands; 5 Department of Religion and Theology, Vrije Universiteit Amsterdam, Amsterdam, The Netherlands

**Keywords:** Religious coping, Surrender, Health, Meaning in life, God representation

## Abstract

Given that surrender to God has been associated with health and well-being in believers, research in this area would benefit from the availability of scales outside the United States, where these were first developed. To this end, we conducted two studies *(N* = 130 and *N* = 574) in Christian samples in the Netherlands to test the psychometric properties of a Dutch translation of the Surrender to God Scale (D-StGS). In addition, the abbreviated Religious Surrender Scale-2 was tested to examine whether the two-item scale would show validity and reliability, as this would benefit research in which there are time (and other) constraints on assessment. Both scales showed adequate to excellent internal consistency, convergent validity with religious coping and religious orientation, and predictive validity; specifically with (I) lower anxiety, stress, and depression symptoms and (II) higher scores on religious behavior and meaning in life. Likewise, both scales were related to (III) more positive, supportive, and ruling, and less passive, anxious, and angry God representations. However, explorative factor analysis of the D-StGS in the first study resulted in two factors (Imitation of God’s will and Peace through God’s will), which were confirmed with confirmatory factory analysis in the second study. It can be concluded that the D-StGS and abbreviated scale are useful for future research in Christian samples.

**Supplementary Information:**

The online version contains supplementary material available at 10.1007/s10943-024-02144-y.

## Introduction

Stressful situations are an unavoidable part of life. The way one copes with such situations has important consequences for health and well-being (e.g., Folkman, 2011). Religion is an important coping resource for many people (Koenig, 2015; Pargament et al., 1998) and can influence a variety of specific coping processes, such as the appraisal of events and psychological responses to those events over the long term (Koenig, 2012; Weber & Pargament, 2014). Surrender to God (StG), a central element of various monotheistic religions (Islam, Christianity, Judaism; Cole & Pargament, 1999), may play an important role in coping with stressors.

The current research consists of two studies aimed at testing the psychometric properties of a Dutch translation of the Surrender Scale (Wong-McDonald & Gorsuch, 2000), because accurate measurement of the StG construct facilitates the refinement of theories of religious coping. StG is a specific form of religious coping, which can be understood as actively letting go of one’s desires and actions to pursue what one believes to be God’s will (Clements & Ermakova, 2012). While many forms of religious coping involve attributing the solution to stressful events to God, believers with high StG emphasize God’s will, and report less impact that factors such as luck and other people may have had on causing their solution (Wong-McDonald & Gorsuch, 2004).

Empirical studies exploring StG as a religious coping strategy show associations with an increased sense of religious well-being (Wong-McDonald & Gorsuch, 2004), religious commitment (Wong-McDonald & Gorsuch, 2000), and feelings of forgiveness, gratitude, and awe (Pugh et al., 2018). StG is also related to mental health, having been associated with reduced anxiety (Clements & Ermakova, 2012) and depression (Pugh et al., 2018). As StG is positively associated with the experience of life’s purpose and satisfaction (Wong-McDonald & Gorsuch, 2004), as part of meaning in life (Martela & Steger, 2016), and negatively associated with worry (Frederick & White, 2015; Knabb et al., 2017), we believe that StG could reduce worry by providing a refocus on meaningful aspects of life that transcend the necessity of control. StG may be psychologically beneficial by redirecting focus to God’s will. It reorients values and approaches away from non-religious aspects of life that may be perceived as threatened or depleted (e.g., money and health), towards potentially accessible and meaningful religious resources (e.g., feeling God’s love and attention, a purposeful life narrative of suffering, and a sense of forgiveness).

Despite the importance of StG for various believers and its potential added value concerning health and well-being, the only measure with adequate psychometric testing is administered in English. Given the overrepresentation of research on StG in the United States, which may differ from other parts of the world, there is a need for greater diversity in research on StG. The current research was designed to address this need. Using a mostly Christian sample in the Netherlands, we conducted two studies investigating the psychometric properties of the translation of the Surrender Scale (Wong-McDonald & Gorsuch, 2000), which we have named the *Dutch Surrender to God Scale* (D-StGS)^1^^1^.

The original scale study by Wong-McDonald and Gorsuch (2000) showed strong positive correlations of StG with intrinsic religiosity (*r* = 0.62), where religion is a central motivation in one’s life and with collaborative religious coping (*r* = 0.49), where coping with stress is achieved through committed religious practice characterized by a deepened relationship with God. In addition, StG had a strong negative correlation with self-directive religious coping (*r* = −0.66), a coping strategy that embraces problem-solving primarily by oneself and without God (Wong-McDonald & Gorsuch, 2000). Therefore, the D-StGS would indicate convergent validity if the scale showed strong positive associations with intrinsic religiosity and collaborative religious coping and a negative association with self-directive religious coping.

Additionally, to improve usability in research on StG, a two-item subset of the Surrender Scale, the abbreviated Religious Surrender Scale-2, has been developed (Clements et al., 2013). The two-item version showed a high correlation with the whole scale and predicted anxiety and stress to the same extent. Besides translation, a shortened scale could improve usability in future studies. Therefore, in addition to the 12-item questionnaire, we tested the abbreviated two-item version for psychometric properties.

The aim of the first study was to assess the reliability, convergent validity, and predictive validity with religious behavior of the D-StGS and the abbreviated scale. Additionally, we examined the factor structure of the D-StGS. Consistent to the expectations set by the original full scale (Wong-McDonald & Gorsuch, 2000), these scales were anticipated to show adequate or better internal consistency and adequate convergent validity characterized by medium to strong associations with other religious coping styles that incorporate the experience of God, and a predominantly intrinsic religious orientation. Furthermore, the study sought to establish predictive validity through positive associations with more frequent religious behavior (church attendance and prayer).

In a second study with a larger sample, a confirmatory factor analysis was employed to test the expected one-factor structure (Wong-McDonald & Gorsuch, 2000). In addition, following the theories that StG provides health and well-being benefits, we tested the predictive validity of D-StGS and Religious Surrender Scale-2 concerning the expected negative relationship with anxiety (Clements et al., 2012), depression, and stress (Pugh et al., 2018), and a positive relationship with meaning in life (Wong-McDonald & Gorsuch, 2004).

Besides the providence God representation assessed by Knabb and colleagues (2017), a number of relevant God representation measures have yet to be investigated. Therefore, in the second study, we also examined the relationships between the StG and various affective and cognitive dimensions of God representations (Murken et al., 2011; Schaap-Jonker et al., 2008). Hence, aspects of God representations related to providence (positive feelings and supportive and ruling/punishing actions) were expected to be positively associated with StG. We also hypothesized that God representations opposed to providence, like passivity of God and anxiety and anger towards God, would be negatively associated with StG.

## Study 1

### Method

#### Participants and Procedure

Previous research was conducted among primarily Christian believers (Clements & Ermakova, 2012; Knabb et al., 2017; Wong-McDonald & Gorsuch, 2000). For reasons of comparability and availability, the study focused on a Christian sample. In order to recruit Christian participants for the study, we approached visitors at the open days of a Christian mental healthcare center and invited them to participate. Of these, 130 agreed and provided informed consent. There was a broad range of participant age (18–80 years, *M* = 41.38, *SD* = 15.15), as presented in Table 1. Participants were mostly female (*n* = 71; 55%) and mainly adhered to Protestant denominations (Evangelical Protestantism 48%, General Dutch Protestantism 25%, Reformed Pietists^2^^2^ 10%, Reformed 8.5%, other Christian denominations 7.7% and one Jewish participant 0.8%).

**Table 1:** *Characteristics of the Sample in Study 1 (N* = *130) and Associations with the D-StGS and RSS-2 Score*

Characteristic (range)	% (n)	Mean (SD) (n)	D-StGS^1^	RSS-2^1^
*Demographic*				
Age, years (18—80)		41.4 (15.1) (129)	*r* = .08	*r* = .08
Gender				
Male	54.6 (71)		48.0	7.8
Female	45.4 (59)		48.2	7.8
*Denomination*^*2*^				
Evangelical Protestantism	47.7 (62)		49.4	7.9
General Dutch Protestantism	24.6 (32)		46.8	7.8
Reformed Pietists	10.8 (14)		48.8	7.9
Reformed	8.5 (11)		42.4	6.7
Other Christian denominations	7.7 (10)		48.4	7.9
Jewish	0.8 (1)		60.0	10.0
*Religious coping*				
Collaborative (6—30)		23.2 (4.4) (130)	*r* = .69***	*r* = .54***
Self-directive (6—30)		12.9 (4.7) (130)	*r* = −.64***	*r* = −.52***
Deferring (6—30)		15.9 (4.5) (130)	*r* = .40***	*r* = .22*
*Religious orientation*				
Intrinsic (8—72)		59.5 (8.1) (130)	*r* = .73***	*r* = .62***
Extrinsic-social (3—27)		12.9 (6.0) (130)	*r* = .08	*r* = .05
Extrinsic-personal (3—27)		16.56 (4.6) (130)	*r* = .26**	*r* = .12
*Religious behaviour*				
Church attendance (1—5)		4.6 (0.7) (130)	*r*_s_ = .24**	*r*_s_ = .29**
Prayer frequency (1—5)		4.6 (0.7) (130)	*r*_s_ = .41***	*r*_s_ = .39***

^*^p < 0.05, **p < 0.01, ***p < 0.001. ^1^Pearson correlation (*r*) or, in the absence of linearity, Spearman correlation (*r*_s_), used to determine the association between D-StGS or RSS-2 and continuous variables; Student’s t-test or analysis of variance used to compare average scores across categorical variables. D-StGS = Dutch Surrender to God Scale; RSS-2 = Religious Surrender Scale-2. ^2^Not significant if the participant of Jewish denomination was removed from the analysis

Participants completed the study in a quiet assessment area. It took approximately 20 min to complete the questionnaires on a computer with Google Forms. A research assistant was available to answer potential questions. Participants were offered a five Euro gift card for participating in the study. The Ethics Review Board of the University of Amsterdam approved the study (registration ID: 2017-DP-8043).

#### Measures

*Surrender to God.* The main instrument of this study, the D-StGS, had 12 items from the original full scale (Wong-McDonald & Gorsuch, 2000) with a Cronbach’s α score of 0.94. In line with Clements and colleagues (2013), the second and seventh items of the D-StGS were used for the abbreviated Religious Surrender Scale-2 because these items correlated most highly with the full scale. The English items are presented in Table 2. Both scales used a five-point scale ranging from (1) strongly disagree to (5) strongly agree.

**Table 2:** Results From a Factor Analysis of the Dutch Surrender to God Scale (D-StGS)

D-StGS		Factor loadings		
		Two factors		One factor
		1	2	1
Factor 1				
3	When my solutions to problems are in conflict with God’s alternatives, I will submit to God’s way	**.80**	.24	**.78**
4	Although certain options to problems may seem more desirable, I will give them up if God directs me to do so	**.79**	.13	**.77**
7	***Although I may not see results from my labor, I will continue to implement God’s plans as long as God directs me to do so***	**.71**	.36	**.77**
6	I will select God’s solution to a problem even if it requires self-sacrifice from me	**.68**	.40	**.78**
8	Even though I may not fully understand God’s solution to a problem, I will carry out God’s solution as God directs me to	**.67**	.39	**.76**
2	***When my understanding of a problem conflicts with God’s revelation, I will submit to God’s definitions***	**.64**	.34	**.71**
Factor 2				
10	I seek meaning in my difficulties by surrendering to God’s guidance	.19	**.78**	**.64**
9	When I think about the troubles I’ve had, I can give thanks for God’s using them for God’s purposes	.20	**.74**	**.63**
12	When I am in distress, my hope is renewed when I act in accordance to God’s directions	.29	**.68**	**.67**
11	I choose to be strong in the Lord, even when it means giving up being strong in myself	.34	**.63**	**.67**
1	When I first try to make sense of a problem, I put God’s understanding above my own	.37	.57	**.66**
5	I will follow God’s solution to a problem regardless of what that action may bring	.53	.54	**.76**

*N* = 130. D-StGS = Dutch Surrender to God Scale. The D-StGS is a Dutch translation of the Surrender Scale (Wong-McDonald & Gorsuch, 2000). Item 2 and item 7 in italic and bold summarize the Religious Surrender Scale-2 (Clements et al., 2013). The translated content of the items are presented in the Supplementary Material. Both scales use a five-point scale ranging from (1) strongly disagree to (5) strongly agree. The extraction method was principal axis factoring with varimax rotation. Factor loadings above .60 are in bold

The translation process followed the recommendations of Koenig and Al Zaben (2021), including the back translation (Sperber, 2004). In the first step, the questionnaire was translated into Dutch, and then in the second step, it was translated back into English by another translator who was blinded to the original questionnaire. Finally, both English versions were compared, leading to minimal modifications to avoid unusual religious statements. The Dutch translation of the items is displayed in the Supplementary Material.

*Convergent validity scales.* Following Wong-McDonald and Gorsuch (2000), the D-StGS and abbreviated scale relationships were examined with self-directive, collaborative, and deferring religious coping scales (Alma et al., 2003; Pargament et al., 1988). The three scales all had six items. The self-directive scale (Cronbach’s α = 0.85) described individuals who solve stressful situations without involving God (e.g., ‘I act to solve my problems without God’s help’). Conversely, in the deferring scale (Cronbach’s α = 0.72), individuals waited for an intervention of God before solving stressful situations themselves (e.g., ‘When a situation makes me anxious, I wait for God to take those feelings away’). However, individuals with collaborative (Cronbach’s α = 0.83) religious coping assign themselves and God a shared responsibility to solve the problem (e.g.,’When I have a problem, I talk to God about it in my prayers to decide together what it means’). Each religious coping scale was rated on a five-point Likert scale, ranging from (1) strongly disagree to (5) strongly agree.

Furthermore, the convergent validity of the D-StGS was assessed using the intrinsic religiosity scale (Gorsuch & McPherson, 1989). Individuals with an intrinsic orientation (e.g., ‘My whole approach to life is based on my religion’; Cronbach’s α = 0.78) regard other needs, although important, as less significant compared to their own religious beliefs and principles (Allport & Ross, 1967). However, in alignment with the approach of Wong-McDonald and Gorsuch (2000), extrinsic scales were also incorporated in the analysis for a comprehensive assessment. In an extrinsic orientation, religious beliefs and principles are utilized as an instrument to serve other personal or social interests (Gorsuch & McPherson, 1989). The intrinsic orientation had eight items; both extrinsic-social (e.g., ‘I go to church mostly to spend time with my friends’; Cronbach’s α = 0.88) and extrinsic-personal (e.g., ‘I pray mainly to gain relief and protection’; Cronbach’s α = 0.68) orientations had three items. All orientations are assessed by a nine-point Likert scale, ranging from (1) strongly disagree to (9) strongly agree.

*Predictive validity scales.* For reasons of predictive validity, we used two scales concerning religious behavior. Church attendance was rated on a one-item five-point scale ranging from (1) once a year or less, to (5) at least once a week. Likewise, prayer frequency was measured on a one-item scale, from (1) never, to (5) several times a day.

#### Statistical Analysis

The multiple data analytical strategies of the present study were performed with IBM SPSS Statistics, version 25. The sample characteristics were determined using descriptive statistics, as recorded in Table 1. In addition, also documented in Table 1, the associations between the total D-StGS and the Religious Surrender Scale-2 score with demographic, religious coping, religious orientation, and religious behavior characteristics were examined using Student’s t-test or analysis of variance to compare for categorical variables and Pearson correlations for continuous variables. If the assumption for linearity was not met, a nonparametric Spearman correlation was chosen.

Next, the reliability was determined using the internal consistency of the Cronbach alpha coefficient. It was calculated for the total 12-item D-StGS and the two-item Religious Surrender Scale-2. Also, we tested the Cronbach alpha coefficient of the D-StGS after each item was removed to determine the contribution the item made to the total scale reliability (Koenig et al., 2015). Following Hunsley and Mash (2007), we considered internal consistency to be *adequate* in the range of 0.70–0.79, *good* with 0.80–0.89, and 0.90 and above as *excellent* (Hunsley & Mash, 2007).

Subsequently, the convergent validity of the D-StGS and abbreviated scale was evaluated with Pearson correlations or, in the absence of linearity, Spearman correlation between the two scales and other measurement instruments of religious coping (collaborative and self-directive religious coping) and religious orientation (intrinsic religiosity), as documented in Table 1. Moreover, predictive validity was determined using measures of religious behavior operationalized as church attendance and prayer frequency.

Further, an exploratory factor analysis was conducted using principal axis factoring to examine the internal structure of the twelve items of the D-StGS (cf. Koenig & Al Zaben, 2021). A Bartlett’s Test of Sphericity and Kaiser–Meyer–Olkin test were performed to determine the factorability of the data. In addition, we followed the Kaiser-Guttman rule, in which an eigenvalue of greater than 1.0 determines whether there is an additional factor. Next, in line with Koenig and Zaben (2021), we chose a varimax rotation because we expected only one factor to be present, as in the original full scale (Wong-McDonald & Gorsuch, 2000).

### Results

#### Sample Characteristics and Descriptive Statistics

Descriptive statistics for demographics and each measure are presented in Table 1. Notably, participants reported a high level of church attendance (once a week or more 69%, three times/twice a month 23%, less 8%) and a high frequency of prayer (several times a day 65%, every day 29%, less 6%).

#### Exploratory Factor Analysis

Factor analytic validity of the D-StGS was determined using principal axis factoring with varimax rotation on the twelve items. According to the Kaiser-Guttman rule, two factors explained 58.4% of the variance. Following the criteria of Kaiser and Rice (1974), sampling adequacy for the analysis was overall ‘marvelous’ (KMO = 0.91), and for individual items ‘meritorious’ (greater than 0.85). The Bartlett’s test of sphericity was acceptable (χ^2^ = 895.5; *p* < 0.001).

The items belonging to the first factor seemed to emphasize the *Imitation* of God’s will (Cronbach’s α = 0.89). The second-factor content highlighted *Peace* through God’s will (Cronbach’s α = 0.83). We found that item 5 in the D-StGS had similar loadings for both factors indicating that the item should be removed. Although there was a small sample size, the factor solution was considered reliable because each factor had four or more factor loadings greater than 0.6 (Guadagnoli & Velicer, 1988).

Nevertheless, given our hypothesis, we ran the analysis again post-hoc with a fixed one-factor solution. The factor loadings in a one-factor solution were above 0.63 on all items, explaining 53.5% of the variance. Table 2 shows the one-factor and two-factor solutions with factor loadings after rotation.

#### Reliability

The StG scales had a Cronbach alpha coefficient of 0.92 (95%; CI 0.90—0.94) for the D-StGS and 0.73 (95%; CI 0.62—0.81) for the Religious Surrender Scale-2. Deleting each item of the D-StGS individually to evaluate how removing the item influenced the D-StGS’s reliability had little effect because all Cronbach alpha coefficients were 0.91. The factors from the exploratory factor analysis had a Cronbach alpha coefficient of 0.90 on the first factor (Imitation) and 0.87 on the second factor (Peace). Given the strong correlation between the two factors (*r* = 0.66; *p* < 0.001) and for practical purposes, we used the one-factor solution in further analysis.

#### Surrender to God Scales

The average StG score on the D-StGS was 48.1 (*SD* = 7.7; range 12 to 60), and on the Religious Surrender Scale-2 was 7.8 (*SD* = 1.5; range 2 to 10). There were no significant differences on the StG scales related to gender or age, as documented in Table 1. One participant of the Jewish denomination reported the maximum score (60) on the D-StGS and Religious Surrender Scale-2 (10). When this participant was removed from the analysis, limiting the analysis to Christian denominations, there was a non-significant difference. In Christian denominations, participants belonging to Evangelical Protestantism scored highest on the D-StGS (*M* = 49.4; *SD* = 5.6) and Religious Surrender Scale-2 (*M* = 7.9; *SD* = 1.2), and those belonging to a Reformed background scored lowest (*M* = 42.4; *SD* = 11.7 and *M* = 6.7; *SD* = 2.3, respectively). A large positive significant correlation existed between the abbreviated scale and the total D-StGS (*r* = 0.85; *p* < 0.001).

#### Validity

As evidence for convergent validity, a significant large correlation was found between less self-directive religious coping and the StG score on the D-StGS (*r* = -0.64; *p* < 0.001) and Religious Surrender Scale-2 (*r* = −0.52; *p* < 0.001), as shown in Table 1. Conversely, significant large correlations were found between D−StGS and more intrinsic religious orientation (*r* = 0.73; *p* < 0.001) and collaborative religious coping (*r* = 0.69; *p* < 0.001). Likewise, significant large correlations were found between the abbreviated scale and more intrinsic religious orientation (*r* = 0.62; *p* < 0.001) and collaborative religious coping (*r* = 0.54; *p* < 0.001). Notably, a small correlation was found between the extrinsic-personal orientation and more StG measured with the D-StGS (*r* = 0.26; *p* = 0.003) but not with the Religious Surrender Scale-2.

Further, predictive validity was demonstrated with religious behavior by small correlations between more church attendance and the D-StGS (*r*_s_ = 0.24; *p* = 0.006) and Religious Surrender Scale-2 (*r*_s_ = 0.29; *p* = 0.001). Similarly, prayer frequency showed a medium relationship with the D-StGS (*r*_s_ = 0.41; *p* < 0.001) and abbreviated scale (*r*_s_ = 0.39; *p* < 0.001).

## Study 2

### Method

#### Participants and Procedure

A total of 587 participants were able to complete the questionnaires through an online survey on various websites, such as a Christian research institute or news platform. The sample was also part of another study (Oudijn-van Engelen et al., 2022). The sample had a mean age of 44.0 years (*SD* = 14.8), the majority of whom were women (71.2%) and described themselves as Christian (98.6%).

After providing informed consent, participants filled in the questionnaires without financial compensation. The study was approved by the scientific research committee belonging to the Centre for Research and Innovation in Christian Mental Health Care, Eleos/De Hoop (registration ID: RvB/000351/0699/AH).

#### Measures

*Surrender to God.* The second study used the full-scale D-StGS (Cronbach’s α = 0.94). In addition, analyses were conducted with the abbreviated Religious Surrender Scale-2 (Cronbach’s α = 0.74), as described in Study 1, to measure surrender to God.

*God representations.* The 34 items of the Questionnaire God Representations (Schaap-Jonker & Vrijmoeth, 2023) were used to measure three affective or experiential and three cognitive or doctrinal dimensions of God representations.

The affective dimension consisted of the scales: Positive feelings towards God (Cronbach’s α = 0.92) with nine items (e.g., ‘When I think of God I experience love’), Anxiety towards God (Cronbach’s α = 0.88) with five items (e.g., ‘When I think of God I experience fear of being not good enough’), and Anger towards God (Cronbach’s α = 0.83) with four items (e.g., ‘When I think of God I experience disappointment’).

The cognitive dimension assessed the scales: Supportive actions of God (Cronbach’s α = 0.91) with ten items (e.g., ‘God protects me’), Ruling/punishing actions of God (Cronbach’s α = 0.76) with four items (e.g., ‘God rules’), and Passivity of God (Cronbach’s α = 0.72) with two items (e.g., ‘God leaves people to their own devices’). All scales were rated by participants on a five-point scale ranging from (0) strongly disagree to (4) strongly agree.

*Well−being and health.* The Depression Anxiety Stress Scales−21, a short version of the 42-item version (Antony et al., 1998; Lovibond & Lovibond, 1995), assessed the scales of Depression (Cronbach’s α = 0.93), Anxiety (Cronbach’s α = 0.87), and Stress (Cronbach’s α = 0.91). All scales had seven items like ‘I found it difficult to work up the initiative to do things’ for Depression, ‘I tended to over-react to situations’ for Stress, and ‘I felt scared without any good reason’ for Anxiety. Participants gave responses on a four-point scale ranging from (0) did not apply to me at all, to (3) applied to me very much or most of the time.

Furthermore, the Meaning in Life Questionnaire assessed meaning in life, which can be described as “the sense made of, and significance felt regarding, the nature of one’s being and existence” (Steger et al., 2006, p. 81). It had two scales measuring the presence (Cronbach’s α = 0.88) and the search (Cronbach’s α = 0.87) for meaning in life. Both scales had five items, such as ‘My life has a clear sense of purpose’ or ‘I am always looking to find my life’s purpose’ respectively. Participants rated the items on a seven-point scale ranging from (1) absolutely untrue to (7) absolutely true.

#### Statistical Analysis

As in Study 1, version 25 of IBM SPSS Statistics was used to perform multiple data analytical strategies. The D-StGS and the Religious Surrender Scale-2 were examined for predictive validity and to control for other sample characteristics. While Pearson correlations tested the relationships between the scales and continuous variables (demographic, health, and well-being), categorical variables were examined using Student’s t-test or analysis of variance.

To compare two structural models of the D-StGS, we used Mplus version 8.10 to perform confirmatory factor analysis with all twelve items. The first model was a unidimensional model with StG as the latent variable. The second was a two-factor model with Peace and Imitation as latent variables. Modification indices and standardized residuals (z-scores) were examined for respecifying the models and comparing fit indices.

## Results

### Confirmatory Factor Analysis

**Fig. 1:**
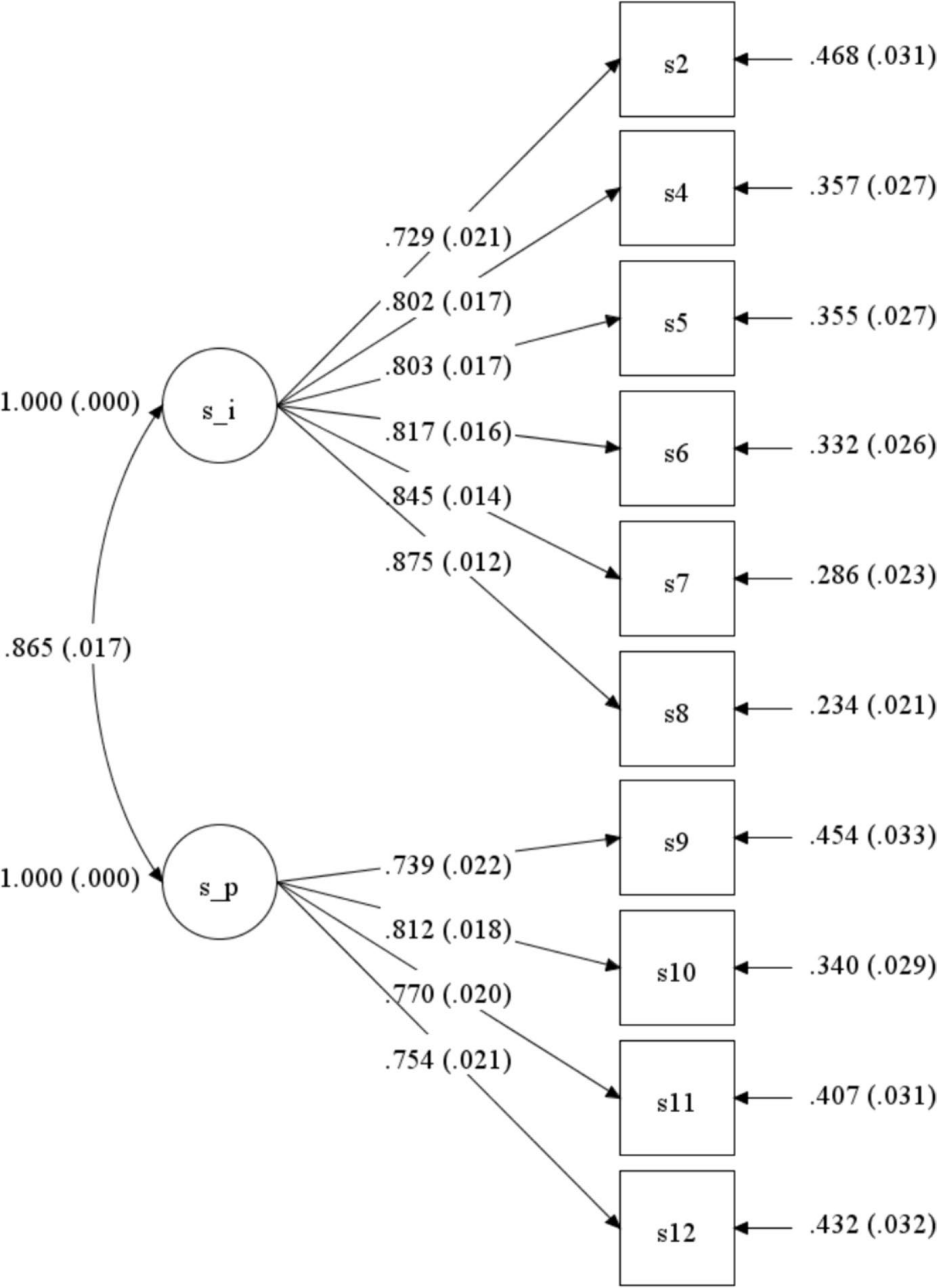
*Diagram of confirmatory two factor model with standardized estimates*. *Note.* s_i = Imitation of God; s_p = Peace through God; s# = item#; Squares represent measured variables, circles represent latent variables

Deleting item 1 and item 3 of the scale addressed multiple fit problems, not item 5, as the analysis in Study 1 would suggest. Fit indices for the one-factor solution suggested model misspecification, while the two-factor model had an acceptable fit, as presented in Table 3. The two-factor model of the D-StGS consists of the scales Peace (Cronbach’s α = 0.85) and Imitation (Cronbach’s α = 0.92) that were highly correlated (*r* = 0.87), which had high factor loadings (Imitation: 0.729-0.875; Peace: 0.739-0.812), as shown in Fig. 1. Given confirmation of the two-factor structure, next to the full D-StGS, the two factors were included in further analysis.

**Table 3:** Model fit indices for the one and two factor Confirmatory Factor Analysis

	χ2	χ2 /df	RMSEA (90% CI)	CFI	TLI	SRMR
One factor CFA	237.83	6.80	0.10 (0.08–0.11)	0.95	0.94	0.04
Two factor CFA	93.60	2.75	0.06 (0.04–0.07)	0.98	0.98	0.02

CFA = Confirmatory Factor analysis; χ2 = Chi Square; RMSEA = Root Mean Squared Error of Approximation; CFI = Comparative Fit Index; SRMR = standardized root mean square residual. Ideal values for fit indices; χ2/df ≤ 5; RMSEA ≤ .06; RMSEA 90% CI must cross 0.05; CFI ≥ .95; TLI ≥ .95; SMSR ≤ .08

### Sample Characteristics and the Surrender to God Scales

The Religious Surrender Scale-2 had a large positive correlation with the total D-StGS (*r* = 0.90; *p* < 0.001). Descriptive statistics for each measure are presented in Table 4. In the second study, the average StG score was 45.9 (*SD* = 9.4; range 12 to 60) on the D-StGS, 15.5 on the factor Peace (*SD* = 3.5; range 4 to 20), 23.2 on the factor Imitation (*SD* = 4.9; range 6 to 30), and 7.5 (*SD* = 1.8; range 2 to 10) on the Religious Surrender Scale-2. While gender and age showed no significant relationships with StG, there were significant relationships between denomination and all the StG scales (e.g., D-StGS: *R*^2^ = 0.163; *p* < 0.001). Of note was, after excluding the eight participants without a Christian denomination only for this analysis, the relation between the StG scales remained significant (e.g., D-StGS: *R*^2^ = 0.084; *p* < 0.001).

**Table 4:** Characteristics of the Sample in Study 2 and Associations with the StG scales

Characteristic (range)	% (n)	Mean (SD) (n)	D-StGS^1^			RSS-2^1^
			Full-scale	Peace	Imitation	
*Demographic*						
Age, years (18—78)		44.0 (14.8) (551)	*r* = .06	*r* = .05	*r* = .04	*r* = .08
Gender						
Female	71.2 (406)		45.6	15.4	23.0	7.4
Male	28.8 (164)		46.6	15.7	23.6	7.7
*Denomination*^*2*^						
Reformed Pietists	36.4 (209)		47.3***	15.8***	24.1***	7.8***
General Dutch Protestantism	21.1 (121)		44.2	15.2	22.2	7.1
Evangelical Protestantism	19.5 (112)		49.2	16.6	24.8	8.0
Reformed	12.2 (70)		44.8	15.1	22.8	7.3
Roman Catholic	5.4 (31)		38.7	13.3	19.2	6.4
Other Christian denominations	2.1 (12)		47.3	15.5	24.3	8.1
Christian, but no denomination	1.9 (11)		43.5	14.9	21.1	7.1
No religion	1.0 (6)		20.0	7.3	9.5	3.2
Humanistic	0.2 (1)		45.0	15.0	24.0	8.0
Jewish	0.2 (1)		26.0	10.0	12.0	4.0
*God Representation*						
Positive (9—45)		36.8 (6.6) (574)	*r* = .60***	*r* = .63***	*r* = .53***	*r* = .51***
Anxious (5—25)		12.8 (5.4) (574)	*r* = −.11**	*r* = −.13**	*r* = −.10*	*r* = −.10*
Angry (4—20)		7.8 (3.6) (574)	*r* = −.31***	*r* = −.35***	*r* = −.27***	*r* = −.25***
Ruling (4 -20)		14.5 (3.9) (574)	*r* = .39***	*r* = .32***	*r* = .40***	*r* = .37***
Supportive (10—50)		43.7 (6.7) (574)	*r* = .66***	*r* = .69***	*r* = .59***	*r* = .56***
Passive (2—10)		3.3 (1.8) (574)	*r* = −.34***	*r* = −.35***	*r* = −.32***	*r* = −.33***
*Well-being and Health*						
Depression (0—21)		5.6 (5.8) (574)	*r*_s_ = −.23***	*r* = −.22***	*r* = −.23***	*r* = −.23***
Anxiety (0—21)		4.1 (4.6) (574)	*r* = −.13**	*r* = −.09*	*r* = −.16***	*r* = −.14**
Stress (0–21)		7.0 (5.5) (574)	*r* = −.21***	*r* = −.20***	*r* = −.20***	*r* = −.21***
Presence Meaning in Life (5—35)		25.9 (6.3) (574)	*r* = .40***	*r* = .42***	*r* = .36***	*r* = .37***
Search Meaning in Life (5—35)		24.8 (6.9) (574)	*r* = .00	*r* = .02	*r* = .00	*r* = −.02

^*^p < 0.05, **p < 0.01, ***p < 0.001. ^1^Pearson correlation (*r*) or, in the absence of linearity, Spearman correlation (*r*_s_), used to determine the association between D-StGS or RSS-2 and continuous variables; Student’s t-test or analysis of variance used to compare average scores across categorical variables. D-StGS = Dutch Surrender to God Scale; RSS-2 = Religious Surrender Scale-2. ^2^The relationship is also significant (*R*^2^ = .084; p < .001) when the analysis is restricted to Christian participants

### Predictive Validity

Table 4 demonstrates the predictive validity of the StG scales concerning well-being and health variables. StG on the full-scale D-StGS was associated with reduced depression (*r* = −0.23; *p* < 0.001), anxiety (*r* = −0.13; *p* = 0.002), and stress (*r* = −0.21; *p* < 0.001) alongside increased meaning in life scores (*r* = 0.40; *p* < 0.001). The same relationships were found with the StG subscales and abbreviated scale.

Additionally, the study found predictive validity in the associations between the StG scales and affective as well as cognitive God representations. For instance, the full-scale D-StGS showed strong relationships with more positive feelings towards God (*r* = 0.60; *p* < 0.001) and with less feelings of anxiety (*r* = −0.11; *p* = 0.008) and anger (*r* = −0.31; *p* < 0.001) towards God. In terms of the cognitive dimension, God’s actions were reported to be less passive (*r* = −0.34; *p* < 0.001), more ruling/punishing (*r* = 0.39; *p* < 0.001), and more supportive (*r* = 0.66; *p* < 0.001). Comparable associations were found with the other StG (sub)scales.

### Discriminant Validity

The absence of associations between StG scales and age, gender, and search for meaning in life, theoretically unrelated variables, supported the discriminant validity of the StG scales.

## General Discussion

In a series of two studies, the aim was to validate the translated D-StGS and abbreviated Religious Surrender Scale-2 (Clements et al., 2013; Wong-McDonald & Gorsuch, 2000) as measures of religious coping. The key findings can be summarized as follows: the one-factor structure of the original full-scale (Pugh et al., 2018; Wong-McDonald & Gorsuch, 2000) was not confirmed, but two (highly correlated) underlying factors were found, which we labeled *Imitation of God’s will* and *Peace through God’s will*. Given their high correlation (0.87), for practical purposes, a single scale score can be used. This combined scale exhibited excellent internal consistency, with convergent validity concerning religious coping and intrinsic religious orientation. Furthermore, there is predictive validity concerning religious behavior, God representations, depression, anxiety, stress, and perceived meaning in life. It can be concluded that the remaining psychometric properties of the D-StGS are consistent with the original full scale, allowing the questionnaire to be used in Dutch-speaking individuals.

In addition, the brief two-item version – the Religious Surrender Scale-2 – showed a strong positive correlation with the full D-StGS and also showed adequate internal consistency. Like the full-scale D-StGS, the two-item scale supported convergent validity through its relationships with religious coping and intrinsic religious orientation. Moreover, there is evidence for predictive validity through the relationships of the abbreviated scale with religious behavior, depression, anxiety, stress, and meaning in life. Although the correlations were weaker than those found for the full scale, using the Religious Surrender Scale-2 could be appropriate in certain research circumstances with little time for assessment.

Furthermore, individuals exhibiting higher levels of StG reported at the same time more meaning in life. This correlation aligns with our hypothesis that StG is linked to a refocus on religiously meaningful resources, such as a sense of God’s love, surpassing the need for control over non-religious resources. Future research should investigate whether this shift constitutes a potential mechanism through which StG reduces stress and worry. Such a causative relationship would be congruent with findings that StG enhances resilience against intolerance of uncertainty (Knabb et al., 2017), a state often related to a perceived lack of control.

However, StG could also lead to mixed or adverse outcomes if helpful religious resources are perceived as blocked. Perhaps with prolonged feelings of being lost or deserving punishment, StG could elicit spiritual struggles. The importance of focusing on religious resources in StG should be studied with a specific inventory of non-religious and religious life areas combined with aspects of health and well-being to examine our suggested mechanism of StG.

### Limitations

An important limitation of the study was the predominant Christian Protestant participant base with high church attendance and prayer frequency, limiting the findings’ generalizability to other religious groups. Future research should involve believers from other monotheistic religions to assess the relevance of StG as a construct and the D-StGS as an instrument.

Further, the study’s cross-sectional design did not allow for the examination of test–retest reliability. Although internal consistency was confirmed, follow-up research has yet to examine the stability of scale responses over time. In addition, the research design prevented conclusions about the potential causal influence of StG. However, a previous randomized control trial with college students showed that the group with an intervention to enhance StG reported less anxiety and more surrender to God during the post-assessment compared to the group without intervention (Knabb & Vazquez, 2018). The same intervention was recently found to be helpful in a patient with substance use disorder and religious and spiritual problems, according to DSM-5 (Seesink et al., 2022a). Follow-up research should reveal whether StG is relevant for patients. Since religious patients frequently mention StG as necessary for recovery, the influence of StG on recovery through a randomized control trial is currently being investigated (Seesink et al., 2022b).

### Conclusion

The two studies provide initial support for the usability of the D-StGS and abbreviated scale for future health research in the Netherlands. As described, the scales presented sufficient internal consistency, convergent and predictive validity regarding religious orientation, coping and behavior, God representations, and aspects of health and well-being.

## Supplementary Information

Below is the link to the electronic supplementary material.

Supplementary file1 (PDF 541 KB)

## Footnotes

1 With the permission of the first author of the Surrender Scale (Wong-McDonald & Gorsuch, 2000), we renamed the translated questionnaire to the Dutch Surrender to God Scale (D-StGS). Moreover, it improves the distinction from another Surrender Scale (Piedmont et al., 2009; Reinert, 1997), which does not measure religious coping.

2 To our knowledge, there is no official English translation of the Dutch denomination, which refer to themselves as ‘bevindelijk’ Reformed. Therefore, this study uses the term ‘Reformed Pietists’ used in another study (Seesink et al., 2022a).
