# Supplementary material for: Psychometric Properties of the Dutch Surrender to God Scale: Relationships with Religious Behavior, God Representation, Well-being, and Health
Source: J Relig Health. 2024 Oct 4;64(2):1399–418. doi: 10.1007/s10943-024-02144-y (PMC11950084; doi:10.1007/s10943-024-02144-y)
Supplement: Supplementary file 1 — Supplementary file1 (PDF 541 KB) [file 10943_2024_2144_MOESM1_ESM.pdf]

Supplementary Material

Psychometric properties of the Dutch Surrender to God Scale: Relationships with religious behavior, God representation, well-being, and health

Questionnaire

The translated items of the Dutch Surrender to God Scale (D-StGS)

	**Statement**
1	Wanneer ik voor het eerst probeer grip te krijgen op een bepaald probleem, plaats ik Gods wil boven die van mezelf.
2	**Wanneer mijn manier van kijken naar een moeilijke situatie niet overeenkomt met Gods Woord, stel ik Gods waarheid boven mijn eigen mening of gevoel.**
3	Als mijn oplossingen voor problemen in conflict zijn met Gods wil, zal ik me onderwerpen aan Gods weg.
4	Ondanks dat sommige oplossingen voor problemen aantrekkelijk lijken, zal ik ze opgeven als God me duidelijk maakt dat te doen.
5	Ik wil ongeacht de gevolgen Gods wijsheid voor het omgaan met moeilijkheden navolgen.
6	In het omgaan met een probleem of moeilijke situatie zal ik Gods weg kiezen ook als dit zelfopoffering van mij vraagt.
7	**Al zou ik geen resultaat van mijn inspanningen zien, toch zal ik doorgaan om Gods plan uit te voeren zolang als God dit van mij vraagt.**
8	Ook al begrijp ik Gods bedoeling voor een probleem niet geheel, toch zal ik Gods wil doen zoals Hij mij dat opdraagt.
9	Als ik denk aan de moeilijkheden die ik heb gehad, ben ik dankbaar dat God deze gebruikt in Zijn plan.
10	Ik zoek betekenis in mijn moeilijkheden door me over te geven aan Gods leiding.
11	Ik zoek mijn kracht in God, ook als dit betekent dat ik het moet opgeven om sterk te zijn in mezelf.
12	Als ik heel onrustig ben, word ik weer hoopvol wanneer ik handel volgens Gods bedoeling.

Note. This questionnaire is the Dutch translation of the Surrender Scale (Wong-McDonald & Gorsuch, 2000). Item 2 and item 7 in bold summarize the Religious Surrender Scale-2 (Clements et al., 2013). Both scales use a five-point scale ranging from (1) strongly disagree [Dutch: Helemaal mee oneens] to (5) strongly agree [Dutch: Helemaal mee eens].
